# Evaluation of radial and ulnar artery blood flow after radial artery decannulation using colour Doppler ultrasound

**DOI:** 10.1186/s12871-021-01538-9

**Published:** 2021-12-10

**Authors:** Li-jia Liu, Hong-mei Zhou, Huan-liang Tang, Qing-he Zhou

**Affiliations:** 1 The Second Clinical Medical College of Zhejiang Chinese Medical University, Hangzhou, China; 2 Department of anesthesiology, The Second Affiliated Hospital of Jiaxing University, Jiaxing, China; 3 Department of ultrasound, Affiliated Hospital of Jiaxing University, No.1882 Zhonghuan South Rd, Jiaxing, 314001 China; 4 Department of anesthesiology and pain medicine, Affiliated Hospital of Jiaxing University, No.1882 Zhonghuan South Rd, Jiaxing, 314001 China

**Keywords:** Catheterization, Ultrasonography, Radial artery, Ulnar artery, Regional blood flow

## Abstract

**Background:**

There is a lack of reports in the literature regarding changes in radial artery blood flow after decannulation. The objective of this study was to investigate changes in radial and ulnar artery blood flow after radial artery decannulation using Doppler ultrasound and to explore the factors that influence radial artery blood flow recovery.

**Methods:**

In current observational study, we used colour Doppler ultrasound to measure the cross-sectional area of the radial (S_R_) and ulnar artery (S_U_) and peak systolic velocity of the radial (PSV_R_) and ulnar artery (PSV_U_) for both hands at four time points in patients with radial artery cannulation: pre-cannulation (T0), 30 min after decannulation (T1), 24 h after decannulation (T2), and 7 days after decannulation (T3). Repeated measures analysis of variance and logistic regression analysis were performed to analyse the data.

**Results:**

Overall, 120 patients were included in the present study. We obtained the following results on the side ipsilateral to the cannulation: compared with T0, the ratio of PSV_U_/PSV_R_ increased significantly at T1 and T2 (*p* < 0.01); compared with T1, the ratio of PSV_U_/PSV_R_ decreased significantly at T2 and T3 (*p* < 0.01); compared with T2, the ratio of PSV_U_/PSV_R_ decreased significantly at T3 (p < 0.01). Female sex (OR, 2.76; 95% CI, 1.01–7.57; *p* = 0.048) and local hematoma (OR 3.04 [1.12–8.25]; *p* = 0.029) were factors that were significantly associated with the recovery of radial artery blood flow 7 days after decannulation.

**Conclusions:**

There was a compensatory increase in blood flow in the ulnar artery after ipsilateral radial artery decannulation. Female sex and local hematoma formation are factors that may affect the recovery of radial artery blood flow 7 days after catheter removal.

## Background

Percutaneous radial artery cannulation is usually used to facilitate perioperative blood pressure management and frequent arterial blood gas determination during major surgery. Because of its consistent anatomic accessibility and ease of cannulation, the radial artery is the regular site for arterial cannulation. Although numerous studies have shown that radial artery cannulation has a low probability of complications [1, 2], some complications can occur, including ischaemia, thrombosis, infection, and pseudoaneurysm, and may have serious consequences [3–5]. The main risk factors for complications were female sex [6], old age [7], use of extracorporeal circulation during surgery [8], hematoma caused by repeated puncture [8], long catheter indwelling time [7], larger diameter of arterial catheter [6], and wrist circumference [9], among others.

Colour Doppler ultrasound has been used as a screening technique to assess distal arterial circulation [10]. In 1981, Doppler ultrasound was used to evaluate blood flow after decannulation for two different methods of radial artery cannulation [11]. Roter et al. [12] reported that arterial Doppler assessment was necessary before arterial cannulation to ensure radial artery accessibility in patients with a history of dyslipidaemia, hypertension, smoking and peripheral arterial disease.

Although many studies have observed complications related to radial artery cannulation, a large sample size study has not yet been conducted to assess the recovery of blood flow after radial artery cannulation and to identify factors affecting blood flow recovery. This study therefore aimed to investigate changes in radial and ulnar artery blood flow using Doppler ultrasound after radial artery decannulation and to explore the factors that influence radial artery blood flow recovery.

## Methods

### Participants and design

Ethical approval for this study (JXEY-2020ZXFQ009) was provided by the Ethics Committee of the Second Affiliated Hospital of Jiaxing University, Jiaxing, China (Chairperson Prof Ning-hua Jiang) on 12 February 2020. In current study, we enrolled 128 patients scheduled for elective surgery between February and December 2020 with an expected postoperative stay > 7 days. The enrolled patients, who required arterial cannulation for continuous blood pressure monitoring, were aged > 20 years and had an ASA physical status of 1–3. All patients agreed to participate in this study at the time of recruitment and signed an informed consent form. The exclusion criteria included the following: without radial artery cannulation; positive modified Allen’s test; signs of inflammation at the intended puncture site; coagulopathy; arterial disease (such as Raynaud’s disease or thromboangitis obliterans); upper extremity or shoulder surgeries; postoperative stay < 7 days; temporary use of vasoactive medication 30 min before the measurement during the study. Patients with abnormal and curved arteries were also excluded.

All patients fasted for 8–10 h and no preoperative medication was administrated prior to surgery. Intravenous access was established after the patient entered the operating room, and Ringer’s lactate 8–10 mL/kg was preloaded before anaesthesia. Patients’ arms were abducted to 90° and supported by the arm bracket without dorsiflexion of the hands when the patient was placed in a supine position on the operating table. The non-dominant hand was the first choice for the cannulation.

After obtaining a negative result on the modified Allen’s test, ultrasound images were obtained using an ultrasound system with a 13–6-MHz linear transducer (HFL38x/13- 6 MHz Transducer FUJIFILM Sonosite, Inc. Bothell, WA 98021 USA) with a small parts imaging capability. Vessel compression was avoided, and oblique vessel images were observed using conductive gel. Regarding intravenous access, we selected the ipsilateral cannulation position beside the radial styloid where a prominent radial artery pulsation was felt. We placed the probe perpendicular and 1 cm proximal to the marked point using B-mode ultrasound. The cross-sectional area of the radial and ulnar arteries, as well as the subcutaneous distance of the radial artery, were measured for both hands. The probe slowly turned to the long axis and switched the mode of colour Doppler ultrasound to observe the radial and ulnar arteries. The Doppler sample gate width was placed in the middle of the blood flow without encompassing the vessel walls, allowing for slight movement. The angle of insonation was adjusted and maintained at 50–60°. The peak systolic velocity was measured directly from a longitudinal duplex Doppler spectral wave form from the central axis of the vessels. A measuring tape was used to measure the wrist circumference snugly through the puncture point. Blood pressure was measured using a portable sphygmomanometer, and the pulse rate was also measured.

Before radial artery cannulation, patients were prepped for an arterial line in the standard sterile fashion with the patient in the supine position, wrist extended to 45°, and hand fixed with adhesive tape. 0.5–1.5 mL of 2% lidocaine was injected subcutaneously for local anaesthesia. A 20-G catheter (20GA 1.88 IN, 1.1*48 mm, flow 42 mL/min, BD Angiocath™, USA) was inserted, guided by sustained palpation and by monitoring the filling of the catheter reservoir. Transfixing method was used. Once the needle reservoir was full, the posterior arterial wall was also punctured. Subsequently, the needle was slightly withdrawn, followed by slow withdrawal of the catheter, until the catheter tip was in the arterial lumen, which was verified by blood in the catheter. Thereafter, the remaining catheter was inserted. The catheter was then securely fixed. Ultrasound guidance is also an option for the cannulation of radial artery. Arterial catheters were connected to pressure transducers and were manually flushed intermittently with heparin (4 U/ml) saline during operation. Unnecessary manipulation or stimulation of the arterial walls was avoided. Surgery was performed under general or regional anaesthesia. The catheter was removed after surgery, and pressure was applied to the cannulation site to stop the bleeding.

Blood pressure was maintained within 20% of baseline for the duration of the investigation with intravenous fluid infusions, varying anaesthetic doses, and vasoactive drugs. We excluded patients who temporarily administered vasoactive drugs 30 min before the measurement of cross-sectional area and peak systolic velocity of the artery during the surgery. Vasoactive drugs mainly include adrenergic receptor agonists or antagonists and nitrates. Patients with intraoperative bleeding > 1000 mL or postoperative hemodynamic instability were also excluded from the analysis.

### Measurements

The cross-sectional area of the radial artery (S_R_), the cross-sectional area of the ulnar artery (S_U_), peak systolic velocity of the radial artery (PSV_R_), and peak systolic velocity of the ulnar artery (PSV_U_) of both hands were measured at four different time points: before anaesthesia (T0), 30 min after catheter removal (T1), 24 h after catheter removal (T2), and 7 days after catheter removal (T3). At each time point, the parameters were measured three times consecutively, and the values were averaged. Blood pressure and heart rate were recorded at the time of each ultrasound measurement. Blood pressure was measured using a blood pressure monitor cuff. The depth from the skin to the artery was recorded using ultrasound. The number of cannulation attempts, complications (including hematoma and thrombosis), ultrasound-guided arterial cannulation, and indwelling time of the catheter were recorded during the procedure. The number of cannulation attempts was defined as the number of radial artery punctures. A hematoma was defined as a solid swelling of clotted blood surrounding the artery confirmed by ultrasound, and thrombosis was defined as local coagulation or clotting of blood in the artery confirmed by ultrasound. All ultrasound images were collected by the same anaesthesiologist. The anaesthesiologist received strict training prior to data collection, who had at least 50 cases of vascular ultrasound examination experience. Patient characteristics, complications, and special conditions were also recorded.

We defined radial artery blood flow as not fully restored if the ratio of PSV_U_/PSV_R_ increased by 15% at T3 compared with that at T0 on the side ipsilateral to the cannulation. Logistic regression was used to analyse the relationship between the recovery of blood flow in the radial artery at T3 and independent variables, including the following: sex, age, body mass index, haematocrit, blood platelet, erythrocyte sedimentation rate, fibrinogen, triglyceride, vascular calcification or plaque, chronic diseases (such as hypertension, diabetes, coronary heart disease, cerebrovascular diseases), wrist circumference, number of cannulation attempts, and duration of indwelling catheter.

### Sample size

The sample size for this study was calculated using the PASS 15 program. In the current study, we aimed to detect a 15% difference in the ratio of PSV_U_/PSV_R_ in an individual overtime on the side ipsilateral to the cannulation. We detected interaction effects of the same magnitude as the within factor and analysed the data using a Geisser-Greenhouse-corrected F test. Our preliminary experiments showed that the average ratio of PSV_U_/PSV_R_ was 1.15, with a standard deviation of 0.25, and an autocorrelation between adjacent measurements of 0.75 on the same individual. We assumed that the first-order autocorrelation adequately represented the autocorrelation pattern. A 15% increase is 1.3225 for a PSV_U_/PSV_R_ ratio of 1.15. We used time means of 1.15, 1.33, 1.25, and 1.16. A total of 117 patients were needed, including 15% dropout, to achieve 90% power and a significance level of 0.05. We enrolled 128 patients in the current study.

### Statistical analysis

All data were analysed using SPSS v25.0 (IBM, Armonk, NY, USA). Continuous variables are presented as mean ± SD, and the median is presented with a 25–75 percentile range as appropriate; categorical variables are presented as n (%).

The differences in mean arterial pressure, heart rate, S_R_, S_U_, PSV_R_, PSV_U_, and ratio of PSV_U_/PSV_R_ between T0 and T3 were analysed by repeated measures analysis of variance, followed by the Bonferroni (B) test, when repeated measures analysis of variance was significant. Logistic regression was used to analyse the relationship between the ratio of PSV_U_/PSV_R_ on the side ipsilateral to the cannulation 7 days after decannulation and independent variables, including sex, age, body mass index, haematocrit, blood platelet, erythrocyte sedimentation rate, fibrinogen, triglyceride, vascular calcification or plaque, hypertension, diabetes, coronary heart disease, cerebrovascular diseases, wrist circumference, number of cannulation attempts, ultrasound-guided arterial cannulation, local hematoma, and indwelling time of the catheter. Statistical significance was set at *p* < 0.05.

## Results

**Fig. 1:**
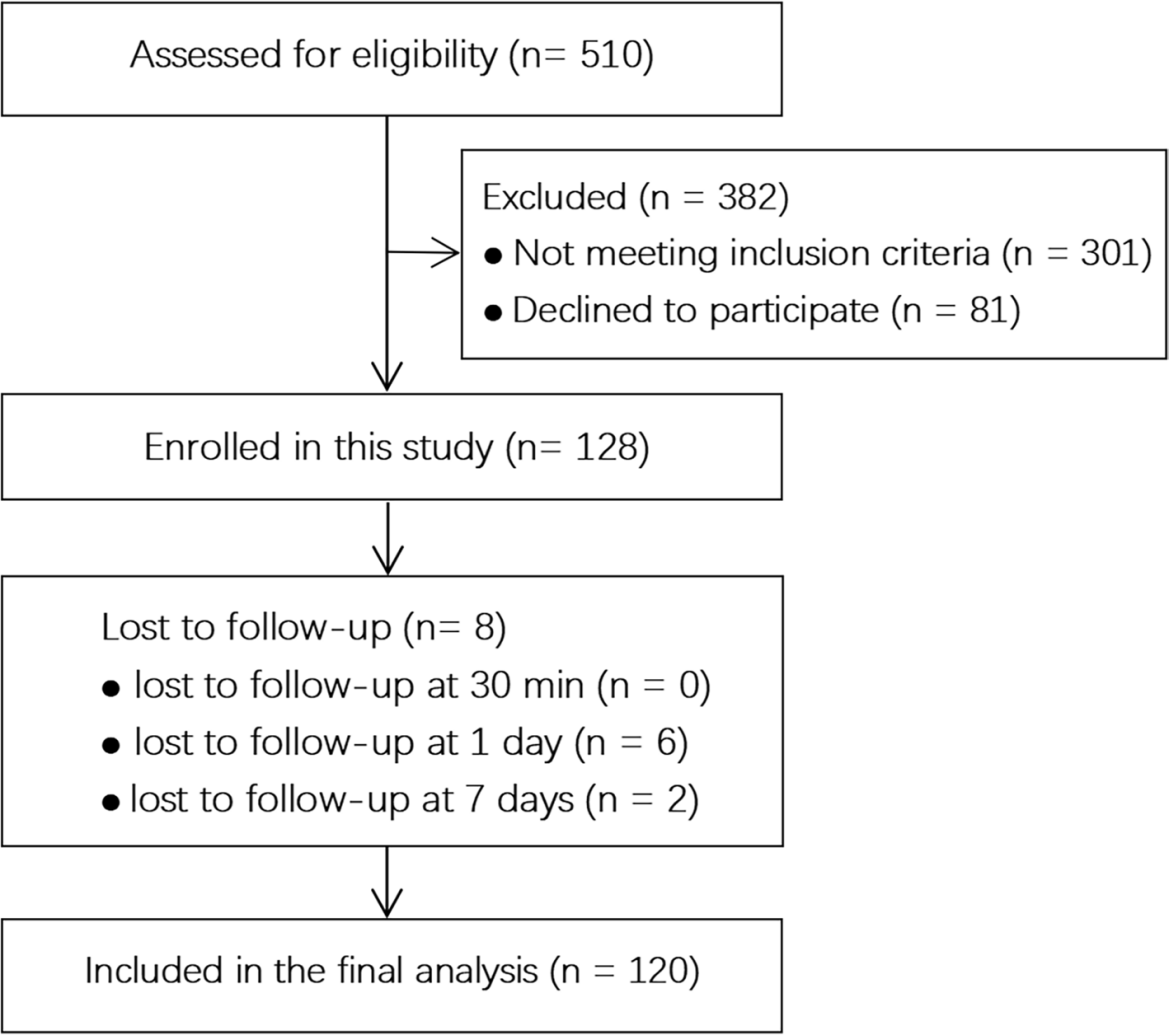
Flow chart of patient enrollment

Initially, 128 patients were enrolled in the current study; however, 8 patients were lost to follow-up. Therefore, 120 patients were ultimately included in the analysis. The descriptive variable characteristics and puncture-related information of the patients are shown in Fig. 1 and Tables 1 and 2.

**Table 1:** Descriptive variable characteristics of the patients (*n* = 120)

Parameters	Value
Age (yrs.)	64.6 ± 11.5
Gender (male/female)	53/67
BMI (kg/m^2^	24.1 ± 3.7
HCT (%)	40.3 ± 4.9
PLT (×10^9^/L)	189.1 ± 57.1
ESR (mm/h)	8.5 [0,19.8]
Fbg (mg/dL)	3.4 ± 0.9
TG (mmol/L)	1.9 [0.9,1.7]
Vascular calcification or plaque* (n, %)	56 (46.7%)
Chronic diseases	
Hypertension (n, %)	53 (44.2%)
Diabetes (n, %)	12 (10.0%)
Coronary heart disease (n, %)	4 (3.3%)
Cerebrovascular diseases (n, %)	5 (4.2%)

*Measurement of carotid and femoral arteries using colour Doppler ultrasound

**Table 2:** Cannulation-related information of the patients(*n* = 120)

Parameters	Value
Cannulation side (left/right)	74/46
Wrist circumference (cm)	16.2 ± 0.8
Distance from the skin to the radial artery (cm)	0.4 ± 0.1
Attempts of cannulation (n)	1 [1,2]
Ultrasound-guided arterial cannulation (n, %)	20 (16.7%)
Local hematoma (n, %)	40 (33.3%)
Indwelling time of the catheter (h)	4.2 ± 1.5

Compared with T0, the mean arterial pressure decreased significantly at T1(*p* = 0.001); compared with T1, and mean arterial pressure increased significantly at T2 and T3 (*p* = 0.007 and *p* = 0.002), while heart rate did not differ among the four time points (*p* > 0.05) (Table 3).

**Table 3:** The haemodynamic information of the patients (*n* = 120)

Parameters	T0	T1	T2	T3	P
MAP (mmHg)	102.0 ± 11.6	97.4 ± 10.9	100.4 ± 10.7	101.4 ± 10.4	0.002
HR (beats/min)	75.3 ± 10.8	77.6 ± 12.1	76.1 ± 10.1	75.3 ± 9.1	0.118

**Fig. 2:**
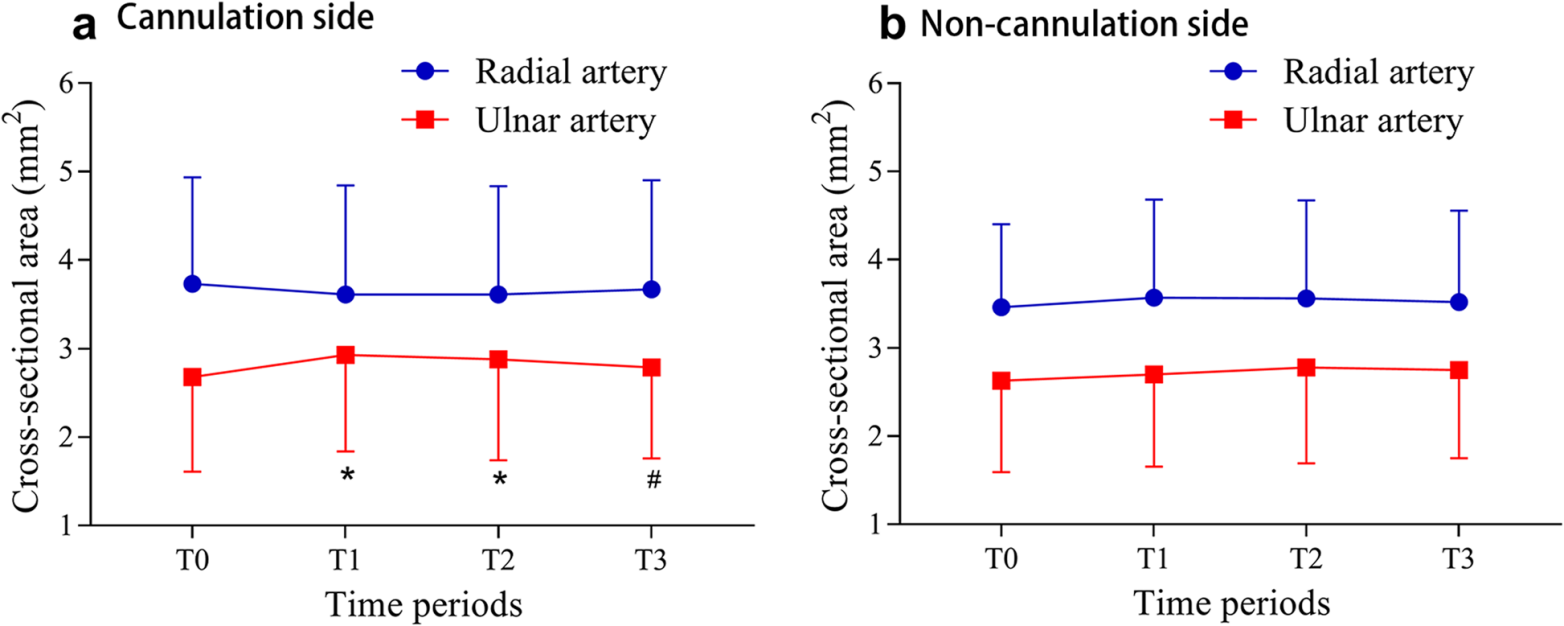
S_U_ and S_R_ on the ipsilateral and contralateral side to cannulation. **a** Compared with T0, the S_U_ increased significantly at T1 and T2 on the side ipsilateral to the cannulation (*p* = 0.001 and *p* = 0.017, respectively). Compared with T1, the S_U_ decreased significantly at T3 (*p* = 0.042), while no difference was found in the S_R_ among the four time points. **b** No difference was found in the S_U_ and S_R_ among the four time points on the side contralateral to the cannulation (all *p* > 0.05)

Compared with T0, the S_U_ increased significantly at T1 and T2 (*p* = 0.001 and *p* = 0.017, respectively) on the side ipsilateral to the cannulation. Compared with T1, the ipsilateral S_U_ decreased significantly at T3 (*p* = 0.042), while no difference was found in the ipsilateral S_R_ among the four time points. Alternatively, no difference was found in the S_U_ and S_R_ among the four time points on the side contralateral to the cannulation (all *p* > 0.05) (Fig. 2).

**Fig. 3:**
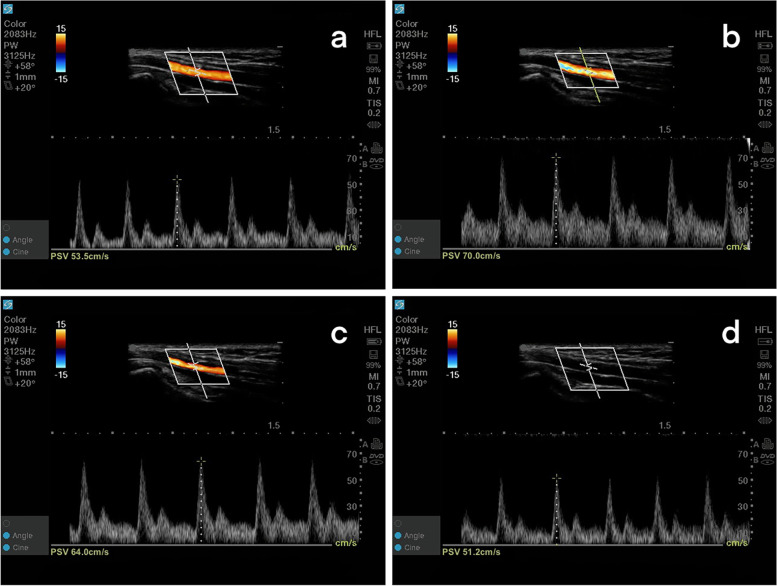
The measurement of PSV_U_ for four timepoints on the side ipsilateral to the cannulation. The PSV_U_ increased from 53.5 cm/s at T0 to 70.0 cm/s at T1 and 64.0 cm/s at T2, and then decreased to 51.2 cm/s at T3

**Fig. 4:**
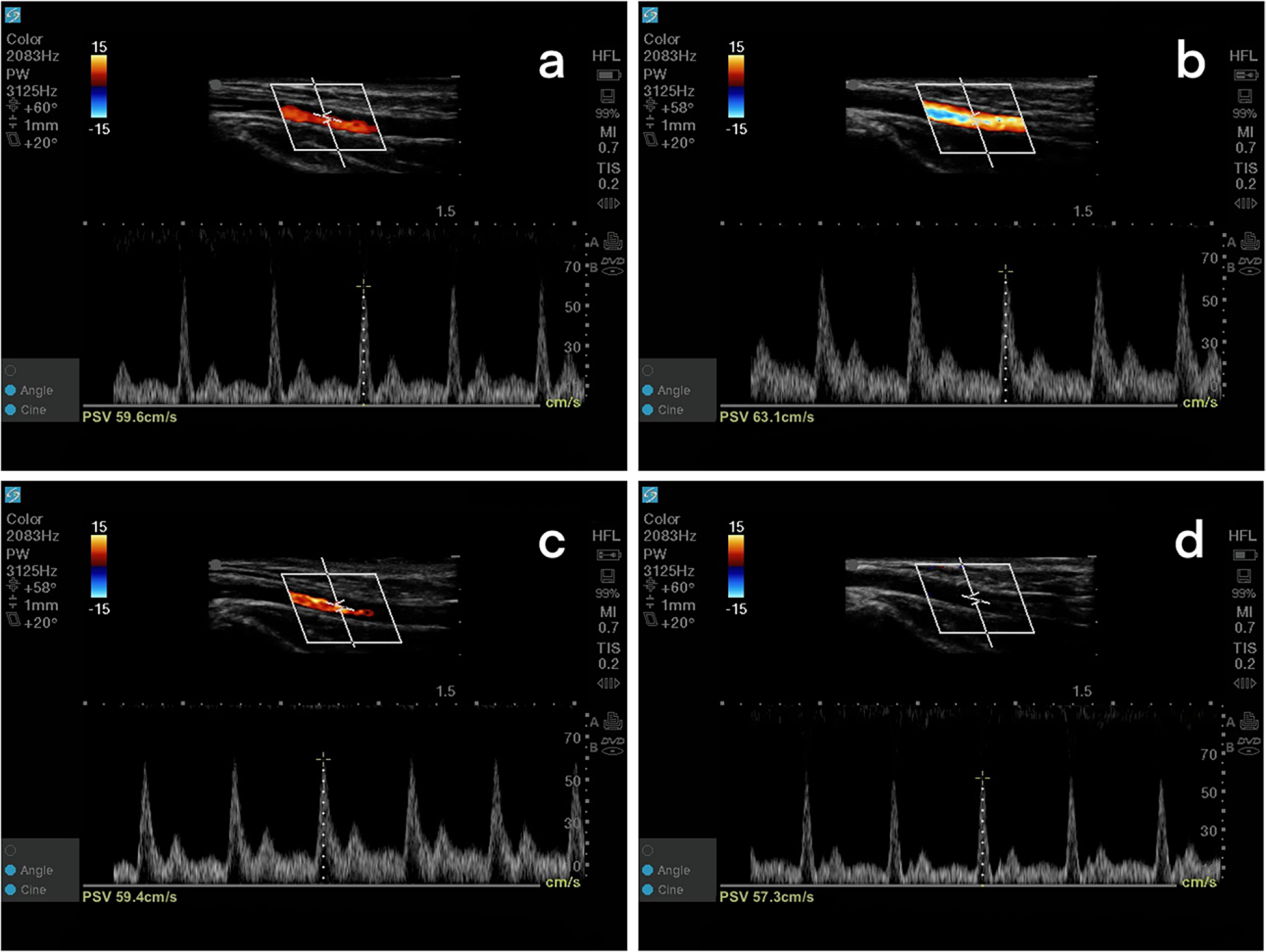
The measurement of PSV_R_ for four time points on the side ipsilateral to the cannulation. The PSV_R_ increased from 59.6 cm/s at T0 to 63.1 cm/s at T1, and then decreased to 59.4 cm/s at T2 and 57.3 cm/s at T3

**Fig. 5:**
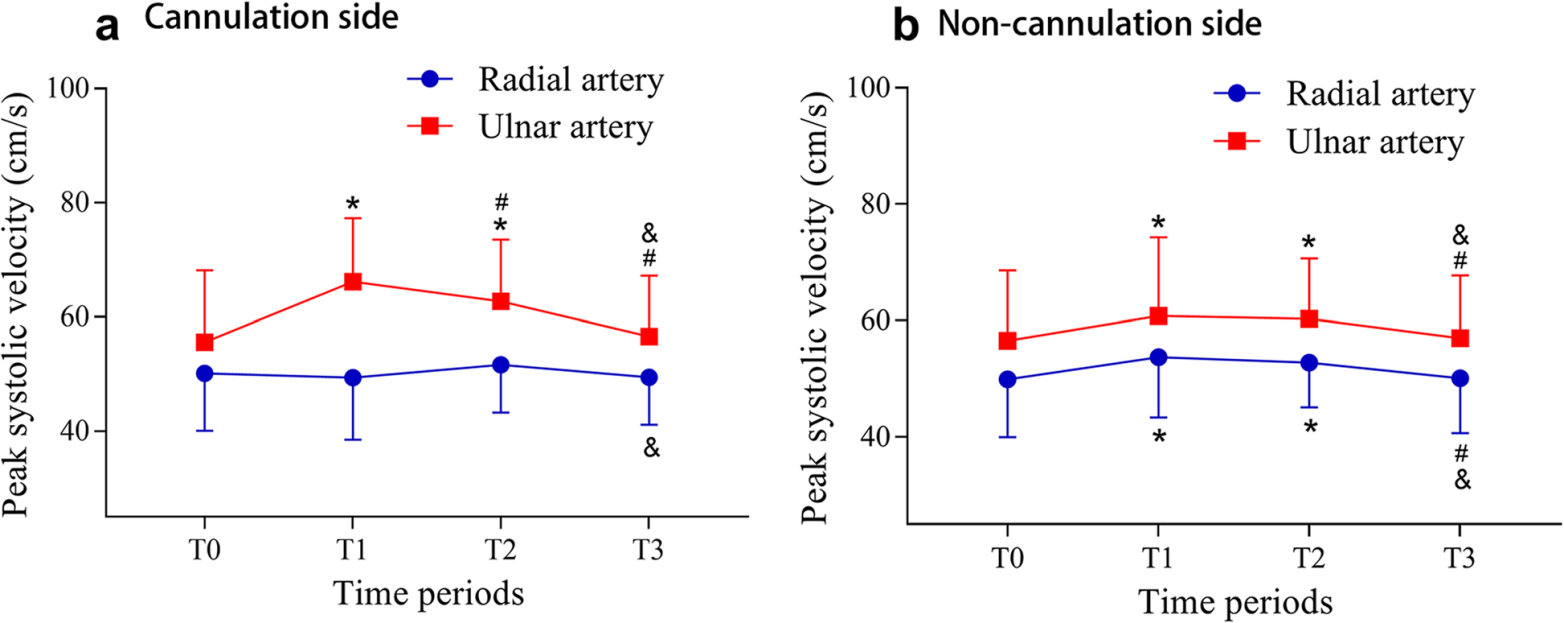
PSV_U_ and PSV_R_ on both the ipsilateral and contralateral sides relative to the cannulation. **a** Compared with T2, the PSV_R_ decreased significantly at T3 on the side ipsilateral to the cannulation (*p* = 0.006). Compared with T0, the PSV_U_ increased significantly at T1 and T2 (both *p* < 0.001); compared with T1, the PSV_U_ decreased significantly at T2 and T3 (*p* = 0.001 and *p* < 0.001, respectively); compared with T2, the PSV_U_ decreased significantly at T3 (*p* < 0.001). **b** Compared with T0, the PSV_R_ increased significantly at T1 and T2 on the side contralateral to the cannulation (*p* < 0.001 and *p* = 0.005, respectively); compared with T1, the PSV_R_ decreased significantly at T3 (*p* < 0.001); compared with T2, the PSV_R_ decreased significantly at T3 (*p* = 0.006). Compared with T0, the PSV_U_ increased significantly at T1 and T2 (*p* = 0.003 and *p* < 0.001, respectively); compared with T1, the PSV_U_ decreased significantly at T3 (*p* = 0.004); compared with T2, the PSV_U_ decreased significantly at T3 (*p* = 0.001)

Compared with T2, the PSV_R_ decreased significantly at T3 (*p* = 0.006) on the side ipsilateral to the cannulation. Compared with T0, the ipsilateral PSV_U_ increased significantly at T1 and T2 (both *p* < 0.001); compared with T1, the ipsilateral PSV_U_ decreased significantly at T2 and T3 (*p* = 0.001 and *p* < 0.001, respectively); compared with T2, the ipsilateral PSV_U_ decreased significantly at T3 (*p* < 0.001). Compared with T0, PSV_R_ increased significantly at T1 and T2 (*p* < 0.001 and *p* = 0.005, respectively) on the side contralateral to the cannulation; compared with T1, the contralateral PSV_R_ decreased significantly at T3 (*p* < 0.001); compared with T2, the contralateral PSV_R_ decreased significantly at T3 (*p* = 0.006). Compared with T0, the contralateral PSV_U_ increased significantly at T1 and T2 (*p* = 0.003 and *p* < 0.001, respectively); compared with T1, the contralateral PSV_U_ decreased significantly at T3 (*p* = 0.004); compared with T2, the contralateral PSV_U_ decreased significantly at T3 (*p* = 0.001) (Figs. 3, 4 and 5).

**Fig. 6:**
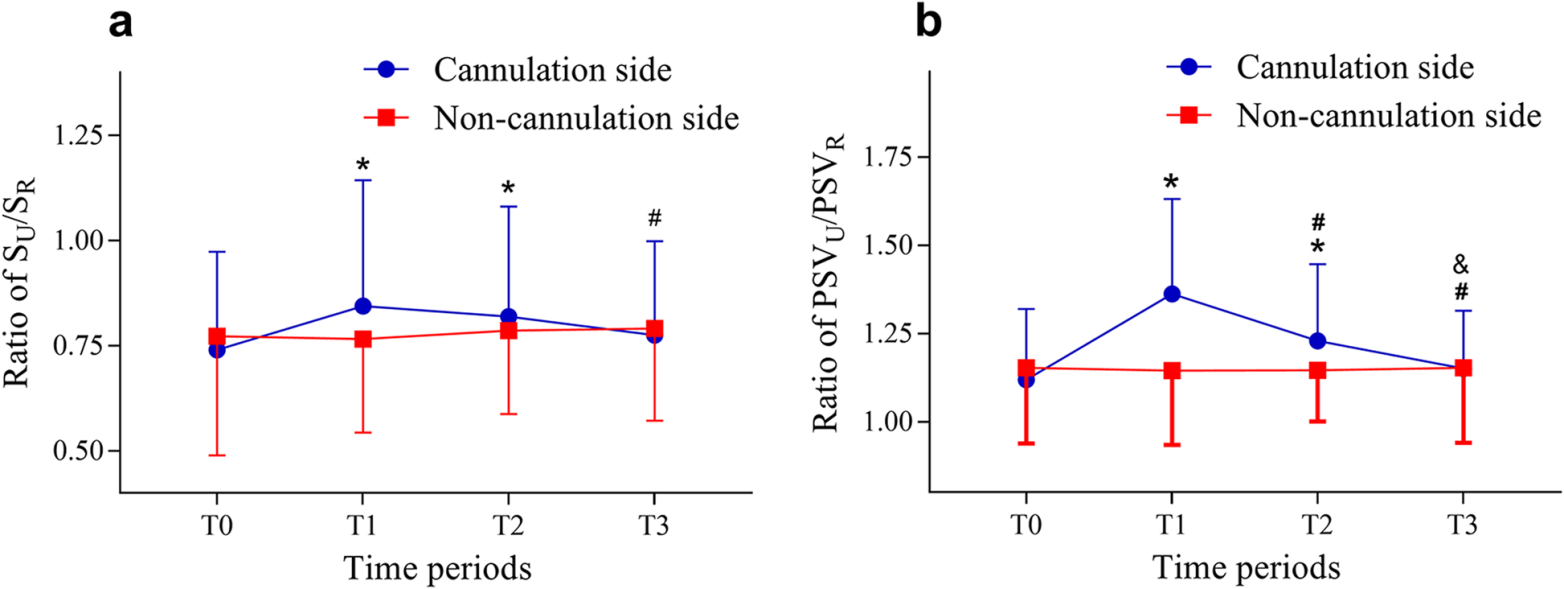
Ratio of S_U_/S_R_ and PSV_U_/PSV_R_. **a** Compared with T0, the ratio of S_U_/S_R_ increased significantly at T1 and T2 on the side ipsilateral to the cannulation (*p* < 0.001 and *p* = 0.002, respectively). Compared with T1, the ratio of S_U_/S_R_ decreased significantly at T3 (*p* = 0.01). No difference in the ratio of S_U_/S_R_ was found among the four time points on the side contralateral to the cannulation. S_U_: cross-sectional area of ulnarartery; S_R_: cross-sectional area of radialartery. **b** Compared with T0, the ratio of PSV_U_/PSV_R_ increased significantly at T1 and T2 on the side ipsilateral to the cannulation (both *p* < 0.001); compared with T1, the ratio of PSV_U_/PSV_R_ decreased significantly at T2 and T3 (both *p* < 0.001); compared with T2, the ratio of PSV_U_/PSV_R_ decreased significantly at T3 (*p* < 0.001). No difference in the ratio of PSV_U_/PSV_R_ was observed among the four time points in the side contralateral to the cannulation

Compared with T0, the ratio of S_U_/S_R_ increased significantly at T1 and T2 on the side ipsilateral to the cannulation (*p* < 0.001 and *p* = 0.002, respectively). Compared with T1, the ratio of S_U_/S_R_ decreased significantly at T3 (*p* = 0.01). No difference in the ratio of S_U_/S_R_ was observed among the four time points on the side contralateral to the cannulation. Compared with T0, the ratio of PSV_U_/PSV_R_ increased significantly at T1 and T2 on the side ipsilateral to the cannulation (both *p* < 0.001); compared with T1, the ratio of PSV_U_/PSV_R_ decreased significantly at T2 and T3 (both *p* < 0.001); compared with T2, the ratio of PSV_U_/PSV_R_ decreased significantly at T3 (*p* < 0.001). No difference in the ratio of PSV_U_/PSV_R_ was observed among the four time points on the side contralateral to the cannulation (Fig. 6).

A total of 21 patients presented a PSV_U_/PSV_R_ ratio increased by 15% at T3 compared with that at T0 on the cannulation side. In our logistic regression model on the recovery of blood flow of the radial artery after decannulation, independent variables significantly associated with the recovery of radial artery blood flow were female sex [OR 2.76 (95% CI 1.01–7.57); *p* = 0.048] and local hematoma [OR 3.04 (1.12–8.25); *p* = 0.029].

## Discussion

Our study showed that there was an obvious compensatory increase in blood flow in the ulnar artery after ipsilateral radial artery decannulation. The compensatory increase of the ulnar artery blood flow normalised and the radial artery blood flow recovered to its pre-cannulation value approximately 7 days after decannulation. Female sex and local hematoma formation may influence the recovery of radial artery blood flow after catheter removal.

In the present study, the ratio of PSV_U_/PSV_R_ increased significantly at 30 min and 1 d after decannulation and subsequently reverted to the pre-cannulation value 7 days after decannulation. The increase in the PSV_U_/PSV_R_ ratio was mainly due to the increase in PSV_U_ and a very slight change in PSV_R_. No obvious change in the ratio of PSV_U_/PSV_R_ was observed among the four time points on the side contralateral to the cannulation. The values of PSV_U_ and PSV_R_ changed synchronously, which led to no obvious change in the ratio of PSV_U_/PSV_R_ on the side contralateral to the cannulation. Thus, the increase in the PSV_U_/PSV_R_ value is mainly due to a compensatory increase in blood flow in the ulnar artery after ipsilateral radial artery decannulation.

Many previous studies have also reported the phenomenon of compensatory increase in blood flow in the ipsilateral ulnar artery. In 2001, a study reported that an increase in peak systolic velocity and blood flow in the ulnar artery was found when radial artery compressed at the wrist [13]. There was a dense network of four arches created by the radial and ulnar arteries at the level of the wrist [2]. If the radial artery is occluded, distal perfusion of the capillary bed will decrease. The blood flow in the ulnar artery increases in tandem with an increase in the pressure gradient of the ulnar artery and capillary bed [13]. In 2012, a study by Kim et al [14] reported that radial artery cannulation can lead to a compensatory increase in blood flow in the ulnar artery after approximately 5 min. Two studies published in 1999 and 2006 showed that ulnar artery blood flow increased significantly after radial artery removal [15, 16]. In the current study, the compensatory increase in blood flow in the ulnar artery disappeared approximately 7 days after decannulation. In line with previous studies [13, 14], we also observed decreased blood flow in the ulnar artery in approximately 20% patients after radial artery decannulation.

In the current study, our results also showed that the ratio of S_U_/S_R_ increased significantly at 30 min and 1 d after decannulation and reverted to pre-cannulation values on the side ipsilateral to the cannulation. This was mainly due to the increase in the S_U_ at 30 min and 1 d after decannulation, while no obvious change was observed in the S_R_ at all time points. Brodmann et al. also reported that radial artery harvest led to an increase in the diameter of the ulnar artery (15.7%) and subsequently induced an increase in the S_U_ [17]. No difference in the ratio of S_U_/S_R_ was observed among the four time points because the values of S_U_ and S_R_ changed synchronously on the side contralateral to the cannulation.

In our study on the analysis of the PSV, S_U_ and S_R_, we used the ratio of PSV_U_/PSV_R_ and S_U_/S_R_ and observed that it was more beneficial to use a single value of PSV_U_, PSV_R_, S_U_, or S_R_. These values can be affected by factors such as anaesthetic drugs, which can induce vasodilatation [15], thereby not reflecting their actual trend overtime. At the same time, we also analysed the data on the side contralateral to the cannulation for comparison. To simplify the analysis, we did not perform a bilateral comparison of these values.

In this study, we observed that the ratio of PSV_U_/PSV_R_ increased by 15% after 7 days of decannulation, which reflects an abnormal recovery of radial artery blood flow on the side ipsilateral to the cannulation. We found that a total of 21 patients (17.5%) had a ratio of PSV_U_/PSV_R_ > 15% 7 days after decannulation, which was substantially higher than that reported by Cronin et al. at 5 days (< 5%) [18], and lower than that reported by Sfeir et al. at 7 days (27.5%) [19]. This may be due to different criteria that were used to determine abnormal blood flow. In the current study, the logistic regression model showed that female sex and local hematoma were independent variables associated with the recovery of radial artery blood flow. The S_R_ of females is relatively small, and both the damage to the blood vessel wall after cannulation and local hematoma affected the S_R_, which can be understood based on Poiseuille’s law. Subsequently, the PSV of the radial artery decreased. Currently, ultrasound-guided radial artery puncture is frequently performed [20–22], which can reduce puncture complications, such as a reduced incidence of haematoma [21]. In the current study, our results shown that ultrasound guidance was not a protective factor for independent variables, which may be due to the relatively small number of ultrasound-guided cases.

The current study has some limitations. Blood flow parameters were not obtained every day after decannulation and were only obtained 30 min after catheter removal, 24 h after catheter removal, and 7 days after catheter removal. In addition, in our study, we only compared the peak systolic velocity. The resistance index, end-diastolic velocity, mean volume flow, and so on, were not compared. Further, radial artery cannulation duration in all patients was less than 6 h, and a previous study reported that the risk of vascular complications arising from percutaneous radial artery cannulation increased after 3 days [23].

## Conclusion

In conclusion, the current study demonstrated that there was an obvious compensatory increase in ipsilateral ulnar artery blood flow after radial artery decannulation and returned to its pre-cannulation value 7 days after decannulation. Female sex and local hematoma formation may influence the recovery of radial artery blood flow after catheter removal.

## Data Availability

The datasets used and/or analyzed during the current study are available from the corresponding author on reasonable request.

## Abbreviations

S_R_: Cross-sectional area of the radial artery
S_U_: Cross-sectional area of the ulnar artery
PSV_R_: Peak systolic velocity of the radial artery
PSV_U_: Peak systolic velocity of the ulnar artery
T0: Time point of pre-cannulation
T1: Time point of 30 min after decannulation
T2: Time point of 24 h after decannulation
T3: Time point of 7 days after decannulation
